# Development and Characterization of Nanoparticles for the Delivery of Gemcitabine Hydrochloride

**DOI:** 10.1155/2014/560962

**Published:** 2014-01-27

**Authors:** Rekha Khaira, Jyoti Sharma, Vinay Saini

**Affiliations:** Department of Pharmaceutics, SD College Pharmacy, Barnala Punjab 148101, India

## Abstract

Gemcitabine (2,2-difluorodeoxycytidine) is a deoxycytidine analog, currently being used as a first-choice drug in pancreatic metastatic cancer. Gemcitabine is administered weekly as 30-minute infusion with starting dose ranging from 800 to 1250 mg/m^2^. The aim of the present work was to develop starch nanoparticles (NPs) for the delivery of gemcitabine hydrochloride that could reduce its dose related side effects and may prolong its retention time (24 hrs) for the treatment of pancreatic cancer. Nanoparticles were prepared by emulsification diffusion method with slight modifications. Size and morphology of nanoparticles were investigated. Particles were spherical in shape with slightly rough surfaces. Particle size and polydispersity index were 231.4 nm and 1.0, respectively while zeta potential of blank NPs and drug loaded NPs were found to be −11.8 mV and −9.55 mV, respectively. Percent entrapment efficiency of different formulations was around **∼**54% to 65%. *In vitro* release profile studies showed that around 70%–83% of drug was released from different formulations. Anticancerous cell line studies were also performed in human pancreatic cell lines (MIA-PA-CA-2).

## 1. Introduction 

Pancreatic ductal adenocarcinoma (pancreatic cancer) is a serious malignant tumour having poor prognosis. Every year approximately 200 000 patients die with pancreatic cancer, and death rate is close to incidence. Pancreatic cancer has a very low rate of 5-year survival not more than 3%–5% [1, 2]. For pancreatic cancer, chemotherapy is an important method for combined therapy, but this have poor therapeutic effect [3]. Many studies have been conducted to improve actual treatment by combining radiotherapy or chemotherapeutic agents, but yet none has shown more than a 10-month survival rate [4, 5]. Gemcitabine (2′,2′-difluoro-2′-deoxycytidine, GEM) is a deoxy cytidine analog; as a chemotherapeutic agent, it is a first-choice drug in the treatment of pancreatic cancer, non-small-cell lung cancer, and ovarian, badder, neck, and head cancer [6]. Gemcitabine is highly hydrophilic, having small molecular weight, has short half-life (17 min), and it quickly decomposed to inactive products after administration. A major limit for the use of gemcitabine is represented by its rapid metabolic inactivation (deamination operated by deoxycytidine deaminase) responsible for its short half-life together with its low but still important systemic toxicity [7]. When standard intravenous infusion dose of 1000 mg/m^2^ is given, a patient's plasma GEM concentration decreases to only 0.4 *μ*g/mL 1.0 hr after administration, considerably below the 5 *μ*g/mL optional plasma concentration for cancer cell inhibition. Thus much larger doses are necessary to reach effective plasma concentrations, posing risk of side effects [8]. Many approaches have been tried to overcome these drawbacks and to increase gemcitabine activity. Namely, the synthesis of saturated and monounsaturated C18 and C20 long chain 4-(N)-acyl derivatives and 5I-esters of gemcitabine (Eli-lily patented) elicited an increase of the drug cytotoxic activity [9, 10]. Another approach involved bioconjugation of gemcitabine with poly (ethylene glycol) (PEG) and folic acid moieties, acting as polymeric drug carrier for the active targeting therapy of cancer disease overexpressing the folate receptor [11]. Another successful strategy to ameliorate the biopharmaceutical properties of the hydrophilic compound was the use of colloidal drug carriers, that is, nanoparticles. Nanoparticles with a mean diameter of 10–1000 nm are widely used as a carrier in drug delivery system [12, 13]. Tumor cells, hepatic Kupffer cells, and cells of the mononuclear phagocyte system have higher phagocytic rates for the uptake of nanoparticles than cells of other tissues, thus increasing the distribution of GEM in tumors, liver, and spleen [14].

## 2. Materials and Methods

### 2.1. Materials

Gemcitabine hydrochloride was a gift sample from Emcure pharma Pvt Ltd.; corn starch soluble, polyvinyl alcohol, acetone, disodium hydrogen orthophosphate, and Sodium chloride were purchased from Central Drug House (P) Ltd., New Delhi. Potassium dihydrogen orthophosphate was purchased from RFCL limited, New Delhi, India.

### 2.2. Materials for Cell Lines

In these studies, human pancreatic cell lines (MIA-PA-CA-2) were used. Appropriate culture medium, Sodium bicarbonate (Sigma, cat. number S5761), 10 mM minimal essential medium (MEM), nonessential aminoacid solution (*In vitro* gen, cat. number 11140), 100 mM sodium pyruvate (Hyclone, cat. number SH30239), FBS (PAA Laboratories, cat. number A11-043), 10 mg mL^−1^ bovine insulin in 25 mM HEPES, pH 8.2 (Sigma, cat. number I0516), 2.5% (wt/vol) trypsin solution (Invitrogen, cat. number 15090), 0.5% (wt/vol) phenol red solution (Sigma, cat. number P0290), 0.48 mM versene-EDTA, 0.4% (wt/vol) trypan blue in 0.81% (wt/vol) NaCl, and 0.61% (wt/vol) KH_2_PO_4_ (Sigma, cat. number T8154) were also used.

### 2.3. Reagents

Dimethyl sulfoxide (DMSO; Sigma (cat. number D4540)), Positive control: doxorubicin (Sigma, cat. number D1515), 10% (w/v) TCA, 1% (v/v) acetic acid, 0.057% (w/v) SRB (Fluka, cat. number 86183) in 1% (v/v) acetic acid, and 10 mm unbuffered Tris base solution were used.

## 3. Optimization of Process Parameters

### 3.1. Effect of Polymer Concentration on Particle Size

In this, different concentrations of polymer were used to prepare nanoparticles, while concentration of polyvinyl alcohol was constant. Optimization was done to study the effect of polymer concentration on particle size. Drug-loaded nanoparticles were prepared by emulsion diffusion method.

### 3.2. Effect of Polyvinyl Alcohol Concentrations on Particle Size

Different concentrations of polyvinyl alcohol were used to prepare nanoparticles while keeping the polymer concentration constant. Nanoparticles were prepared by emulsion diffusion method.

### 3.3. Preparation of Nanoparticles

Drug-loaded nanoparticles were prepared by emulsion diffusion method. Starch and drug in different ratios (1 : 1, 1 : 2, 1 : 3, 1 : 4, 1 : 5, 1 : 6, 1 : 7, and 1 : 8) were dissolved in 5.0 mL acetone. This organic solution containing drug and polymer was poured into 4.0 mL of an aqueous phase containing PVA (1.5% w/v). This biphasic system was emulsified with high speed homogenizer at 2000 rpm during 15 min. After that, 10 mL of highly purified water was added to force the complete diffusion of organic solvent to aqueous phase. Finally, organic solvent was evaporated by magnetic stirrer at 35°C. Highly purified water was added to obtain a colloidal solution of nanoparticles with a final volume of 10.0 mL. The final volume of resulted aqueous nanosuspension was collected. Nanosuspension was then centrifuged at 5000 rpm for 30 min. Nanoparticles were collected. Collected nanoparticles were washed three times with purified water and centrifuged. Finally, nanoparticles were collected.

### 3.4. Sterility Testing

All parenteral preparations should be sterile. Sterility studies were carried out to ensure the sterility of finished product. Since it is administered by parenteral route, direct inoculation method was preferred to carry out sterility testing. In this method, the specified quantity of sample under test was drawn aseptically from the containers and transferred to fluid thioglycollate medium (20 mL) and Soybean-Casein digest medium (20 mL), separately. Mixture of nanoparticles with the medium was incubated for not less than 14 days at 30°C–35°C in case of fluid thioglycolate medium and 20°C–25°C in case of Soybean-Casein digest medium. The growth of any microorganisms in the medium was observed [15].

## 4. Characterization of Nanoparticles

### 4.1. Particle Size Distribution and Zeta Potential

The particle size and polydispersity index were performed by dynamic light scattering (DLS) [16]. The size measurement was performed at 250 cat. 900 scattering angle, and it was recorded for 180 s for each measurement. Nanoparticles were characterized with zeta potential (**ζ**) using a zetasizer 4 (Malvern Instruments Ltd., Malvern, UK) [17]. The zeta potential was measured by an aqueous dip cell in an automatic mode. Samples were diluted in ultrapurified water and placed in a capillary measurement cell, with the cell position adjusted.

### 4.2. Scanning Electron Microscopy

Measurement of particle size and information about shape of the particle were obtained using FEG-SEM (JSM-7600 F, Jeol, Tokyo). The samples for SEM were prepared by sprinkling the nanoparticle powder on a double adhesive tape that stucks to an aluminium stub. They were then vaccum-coated for 45 seconds with platinum mixture. The samples were than randomly scanned and photographs were taken randomly [18].

### 4.3. Transmission Electron Microscopy

Distribution of gemcitabine-loaded nanoparticles was observed under Transmission Electron Microscopy, Hitachi (H-7500). One drop of diluted gemcitabine-loaded nanoparticles suspension was deposited on a film-coated copper grid and it was stained with one drop of 2% (w/v) aqueous solution of phosphotungstic acid. Excess of solution was drained off with a filter paper and then grid was allowed to dry for contrast enhancement. The sample was then examined by Transmission Electron Microscopy [19].

### 4.4. Percentage Yield

Nanoparticles were collected and weighed accurately. The percentage (%) yield was then calculated using formula given below [20]:

(1) $$\begin{array}{c} \%\;\text{Yield}=\frac{\text{Mass}\;\text{of}\;\text{nanoparticles}\;\text{obtained}}{\text{Total}\;\text{weight}\;\text{of}\;\text{drug}\;\text{and}\;\text{polymer}}\times 100. \end{array}$$

### 4.5. Encapsulation Efficiency

The amount of gemcitabine entrapped in the nanoparticles was determined by the separation of gemcitabine-loaded nanoparticles from the suspension containing free gemcitabine by centrifugation. The suspension obtained after solvent evaporation was centrifuged, and the amount of free gemcitabine in the supernatant was measured by ultraviolet (UV) spectrophotometer at 268 nm. The amount of drug entrapped into nanoparticles was calculated as the difference between the drug used for the formulation and the amount of drug in the supernatant. The percent of entrapment efficiency was calculated by the following formula [21]:

(2) %  Entrapment  Efficiency =Total  amount  of  drug  added−Nonbound  drug  Total  amount  of  drug  ×100.

### 4.6. Drug Content

A quantity of drug-loaded nanospheres from each batch equivalent to 1.0 mg was added to 50 mL of normal saline and stirred continuously for 2.0 hrs and then the final colloidal suspensions were centrifuged at 2000 rpm at 22 ± 2°C for 0.5 h. The supernatant was analyzed for drug content by measuring the absorbance at 268 nm using UV spectrophotometer [22].

### 4.7. *In Vitro* Drug Release Studies

30 mg of drug-loaded nanoparticles was placed in an USP dissolution test apparatus having basket type stirring element. The basket was covered with cellophane membrane. 900 mL of phosphate buffer solution (pH 7.4) was used as dissolution medium and kept at 37°C. The basket was rotated at a speed of 100 rpm. 5.0 mL of medium was withdrawn at various time intervals of 1.0 hr, 2.0 hrs, 3.0 hrs, 4.0 hrs, 5.0 hrs, 6.0 hrs, 7.0 hrs, 8.0 hrs, 9.0 hrs, 10.0 hrs, 12.0 hrs, and 24.0 hrs, with the help of 5.0 mL pipette and replaced by 5.0 mL of phosphate buffer solution (pH 7.4). The drug content was estimated by UV spectrophotometer at 268 nm [23].

### 4.8. Stability Studies

Stability studies of prepared nanoparticles were carried out, by storing formulation F5 at 4°C ± 1°C and 30°C ±  2°C in stability chamber for 90 days. The samples were analyzed for drug content (ICH Q1A (*R*_2_) 2003) [24].

### 4.9. *In Vitro* Cell Line Studies

#### 4.9.1. Procedure

These studies were performed according to sulforhodamine B (SRB) assay.

#### 4.9.2. Compound Preparation

Sample was dissolved with 100% (v/v) DMSO to 10 mg/mL. For primary screening, dissolved compound was diluted to 1.0 mg/mL using sterile deionized water. IC_50_ was determined to make a twofold serial dilution from 1.0 mg/mL and 15.625 mg/mL in 10% (v/v) DMSO. Compound solution was mixed by pipetting thoroughly several times after each transfer. Six concentrations of positive controls were prepared from 160 to 5.0 mg/mL using twofold serial dilution in 10% (v/v). 10 mL test sample was added in 10% (v/v) DMSO to each compound well of a 96-well tissue-culture plate (in triplicate). 10% (v/v) DMSO was added into each negative-control well. 10 mL of doxorubicin was added in 10% (v/v) DMSO as a positive-control well [25].

#### 4.9.3. Cell Preparation

Prior to performing the assay, medium from cell monolayers was removed and wash the cells once with sterilized PBS. PBS was removed and 0.25% (w/v) trypsin in versene-EDTA was added to evenly cover the cell-growth surface. When the cells start to dissociate, sterilized plastic or glass pipette was used to disperse them from the culture surface with 10 volumes of culture medium containing FBS and mix them to obtain a homogeneous cell suspension. The cell suspension was transferred to a sterilized polypropylene tube. The cell concentration was determined by counting in a hemocytometer chamber under a microscope, using a 1 : 1 mixture of cell suspension and 0.4% (w/v) trypan blue solution. The assay should not be performed if a large portion of the cells looks unhealthy and stains with trypan blue dye. Adjust the cell concentration with growth medium to obtain an appropriate cell seeding density.

#### 4.9.4. Exposure of Cells to Test Compound

190 mL cell suspension was added to the prepared assay plates. Occasionally, mix the cell suspension during plating to ensure an even distribution of the cells. A plate containing only cell suspension in three columns for a no-growth control (day 0) was chosen as control. The plate at 37°C in a humidified incubator with 5% CO_2_ was kept until cell attachment was completed. The remaining assay plates were incubated at 37°C in a humidified incubator with 5% CO_2_ for 72 h.

#### 4.9.5. Cell Fixation and Staining

Without removing the cell culture supernatant, 100 mL cold 10% (w/v) tricarboxlic acid (TCA) was added to each well, and the plates were incubated at 4°C for 1.0 h. These cells were fixed as effectively as at 10% (w/v). Fixation of loosely attached cell lines will require higher TCA concentrations and extended incubation at 4°C. A trial experiment was carried out to determine the optimal fixing conditions before performing an assay with new cell lines. The plates were washed four times with slow-running tap water via plastic tubing connected directly to a faucet and remove excess water using paper towels. The plates were completely dried by using blow dryer and then allowed to air-dry at room temperature (20–250). Add 100 mL of 0.057% (w/v) SRB solution to each well. Plates were left at room temperature for 30 min and then were rinsed four times with 1% (v/v) acetic acid to remove unbound dye. The plates were dried by using blow dryer and then were allowed to air-dry at room temperature.

#### 4.9.6. Analysis of Results

The percentage of cell-growth inhibition was calculated by using the formulae given below. For IC_50_ determination, plot was drown between dose-response curve versus concentration of compound and percent growth inhibition. IC_50_ values can be derived using curve-fitting methods with statistical analysis software or IC_50_ calculation software [25]. Consider

(3) %  of  control  cell  growth =mean  ODsample−mean  ODday0mean  ODneg  contraol−mean  ODday0×100,%  growth  inhibition=100−%  of  control  cell  growth,%  cells  killed=100−mean  ODsamplemean  ODday0×100.

## 5. Results and Discussion

The aim of present study was to prepare and evaluate starch nanoparticles for the controlled drug delivery of gemcitabine hydrochloride. Eight batches of nanoparticles were prepared using emulsion diffusion technique in order to study the process parameters (formulation development and *in vitro* characterization).

Polyvinyl alcohol is a surfactant and stabilizing agent. Size of nanoparticles depends on the concentration of polyvinyl alcohol. When concentration of PVA was increased, particle size was found to be decreased. Polymer concentration also affects the size of nanoparticles. Particle size of NPs was found to be increased on increasing the concentration of polymer. Nanoparticles with smaller size have valuable characteristics such as improved drug delivery, longer circulation in blood, and lower toxicity. Particle size and polydisersity index were 231.4 nm and 1.0, respectively, while zeta potential of blank NPs and drug loaded NPs were found to be −11.8 mV and −9.55 mV, respectively (Table 1).

**Figure 1:**
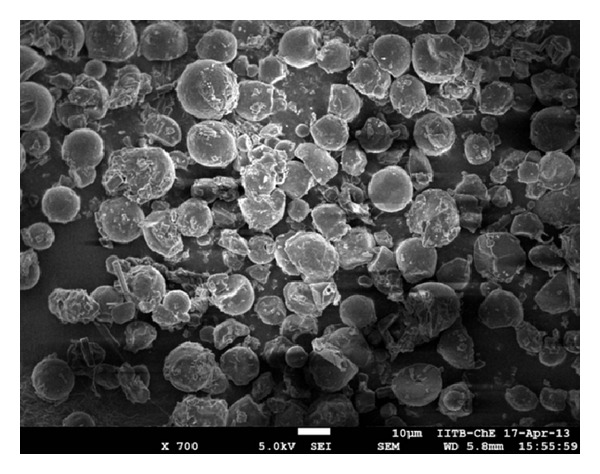
Scanning electron microphotograph (SEM) of gemcitabine hydrochloride loaded starch nanoparticles.

**Figure 2:**
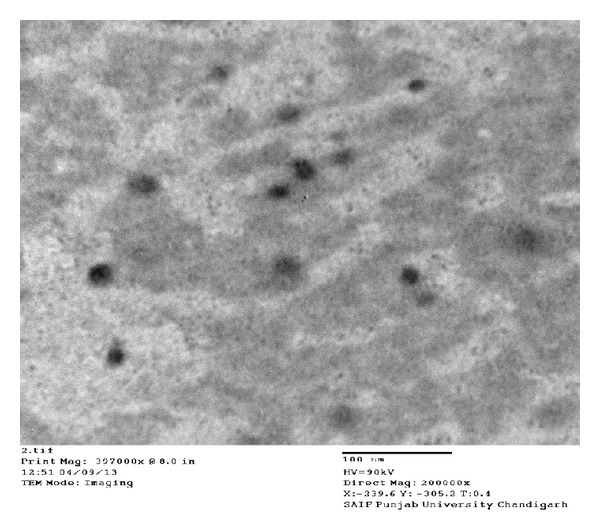
Transmission electron microphotograph (TEM) of gemcitabine hydrochloride loaded starch nanoparticles.

**Figure 3:**
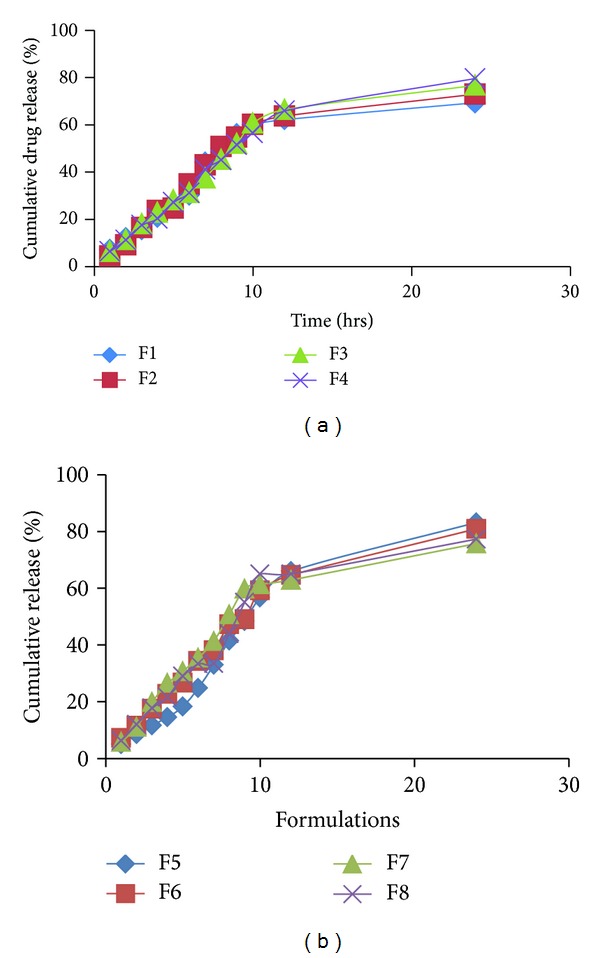
(a) Cumulative % drug release (*in vitro*) for F1–F4. (b) Cumulative % drug release (*in vitro*) for F5–F8.

**Figure 4:**
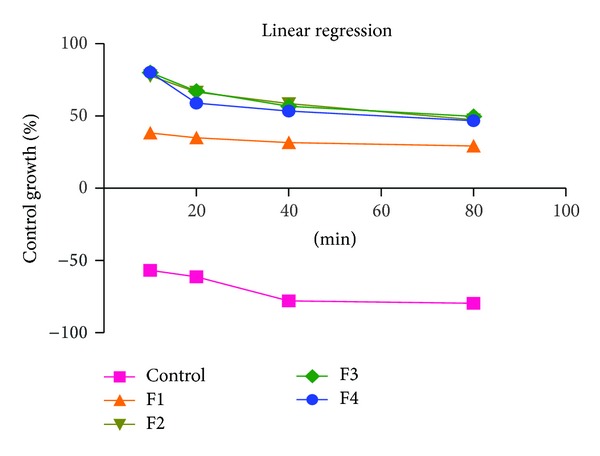
GI 50 (50% of growth inhibition) of averages of three experiments.

During SEM analysis, we found that starch nanoparticles were spherical in size and had slightly rough surface (Figure 1). TEM analysis confirmed that nanoparticles were nanometer in size (Figure 2). Percentage yield of gemcitabine-loaded nanoparticles was from 52 to 87% for different formulations. It can be inferred that, as the concentration of polymer increases, practical yield was also increased. In these studies, we found that around 70% of drug was entrapped in nanoparticles (Figure 3). Drug content for different nanoparticle formulations (F1–F8) was around 70%. It was observed that drug content was increased with increase in drug polymer ratio (1 : 5) (Table 2). After that, drug content was found to be decreased; this might be due to the separation capacity of the polymer. It was observed that, on increasing the concentration of polymer, *in vitro* release was increased up to certain extent, that is, polymer proportion (1 : 5). The cumulative percent release of drug for various formulations (F1–F8) was found to be from 70 to 83% for different formulations. Among all formulations, maximum drug (around 83%) was released from F5 (Table 1 and Figure 3). Burst release of gemcitabine was resultant from nanoparticles at initial stage. It might be due to dissolution of drug crystals on the surface of nanoparticles. Electrostatic interactions between protonated amino residues on gemcitabine and anionic group are responsible for the surface bound interactions involved in the burst release. On observation, we found that maximum *in vitro* release was found to be for F-5 formulation. During sterility testing, we found that there was no evidence of microbial growth when formulations were incubated for not less than 14 days at 30°C to 35°C in case of fluid thioglycolate medium and at 20°C to 25°C in case of Soybean-Casein digest medium. Preparation passes the sterility test. Stability studies were performed according to ICH guideline. In these studies we found that, there was little or no change in parameters (drug content, % cumulative release of drug), when nanoparticles were stored at 4° ± 1°C. At 30° ± 2°C, there was ±5% decrease observed in drug content and ±5% increase in % cumulative drug release. Increase in drug release may attribute to bulk erosion of nanoparticles to some extent during storage. Results concluded that formulation was most stable at 4° ± 1°C. Cell line studies (MIA-PA-C-2) were performed for best formulation (F-5). The anticancer activity of best formulation (F5) was compared with standard. The percentage growth control of cell was determined and found that formulation could not inhibit the growth significantly. Therefore, more experiments (at varying concentrations of drug in nanoparticles) should be performed to establish the potency (efficacy) of starch nanoparticles against various cancers (Figure 4).

## Figures and Tables

**Table 1:** Various physicochemical characterizations of gemcitabine-loaded starch NPs.

S. number	Particle size (nm) (average)	Poly dispersity index	Zeta Potential (mV)		Particle shape	% yield	% entrapment efficiency	Drug content (mg)	*In vitro* drug release in 24 hrs
			Blank NP's	Drug-loaded NP's					
F5	231.4	1.0	−11.8	−9.55	Spherical with slight rough surface	72.5	70.32	69.37	83.13

**Table 2:** % yield, encapsulation efficiency, and drug content of various formulations.

S. number	Formulation	% yield	Encapsulation efficiency	Drug content
1	F1	51.25	53.43 ± 0.42	52.61 ± 0.37
2	F2	59.16	58.14 ± 0.20	56.43 ± 0.28
3	F3	63.75	62.7 ± 0.21	61.18 ± 0.20
4	F4	69.5	66.68 ± 0.32	63.46 ± 0.36
5	F5	72.5	70.32 ± 0.41	69.37 ± 0.37
6	F6	77.8	69.35 ± 0.44	66.68 ± 0.35
7	F7	82.81	67.73 ± 0.36	64.45 ± 0.38
8	F8	86.38	64.33 ± 0.45	59.33 ± 0.39
